# 4-(Naphthalen-1-yl)pyridine

**DOI:** 10.1107/S1600536813014372

**Published:** 2013-06-08

**Authors:** Antje Vetter, Wilhelm Seichter, Edwin Weber

**Affiliations:** a Institut für Organische Chemie, TU Bergakademie Freiberg, Leipziger Strasse 29, D-09596 Freiberg/Sachsen, Germany

## Abstract

In the title compound, C_15_H_11_N, the mean planes of the aromatic moieties are inclined to one another by 72.9 (1)°. The crystal is stabilized by π–π stacking inter­actions between the pyridine rings of inversion-related mol­ecules, with a centroid–centroid distance of 3.772 (2) Å. In addition, C—H⋯π contacts involving an α-C—H group of the pyridine ring and the nonsubstituted ring of the naphthalene unit are observed, giving rise to a herringbone-type supramolecular architecture of the naphthalene moiety being contained in the molecule.

## Related literature

For preparative methods and the characterization of the title compound, see: Miyaura *et al.* (1981 ▶); Broutin & Colobert (2005 ▶); Molander & Beaumard (2010 ▶). For π–π stacking inter­actions, see: James (2004 ▶). For C—H⋯π inter­actions, see: Nishio *et al.* (2009 ▶). For non-classic hydrogen bonds, see: Desiraju & Steiner (1999 ▶). For related structures, see: Boeyens *et al.* (1988 ▶); Fabbiani *et al.* (2006 ▶); Suthar *et al.* (2005 ▶). For aspects of organic crystal engineering, see: Tiekink *et al.* (2010 ▶).
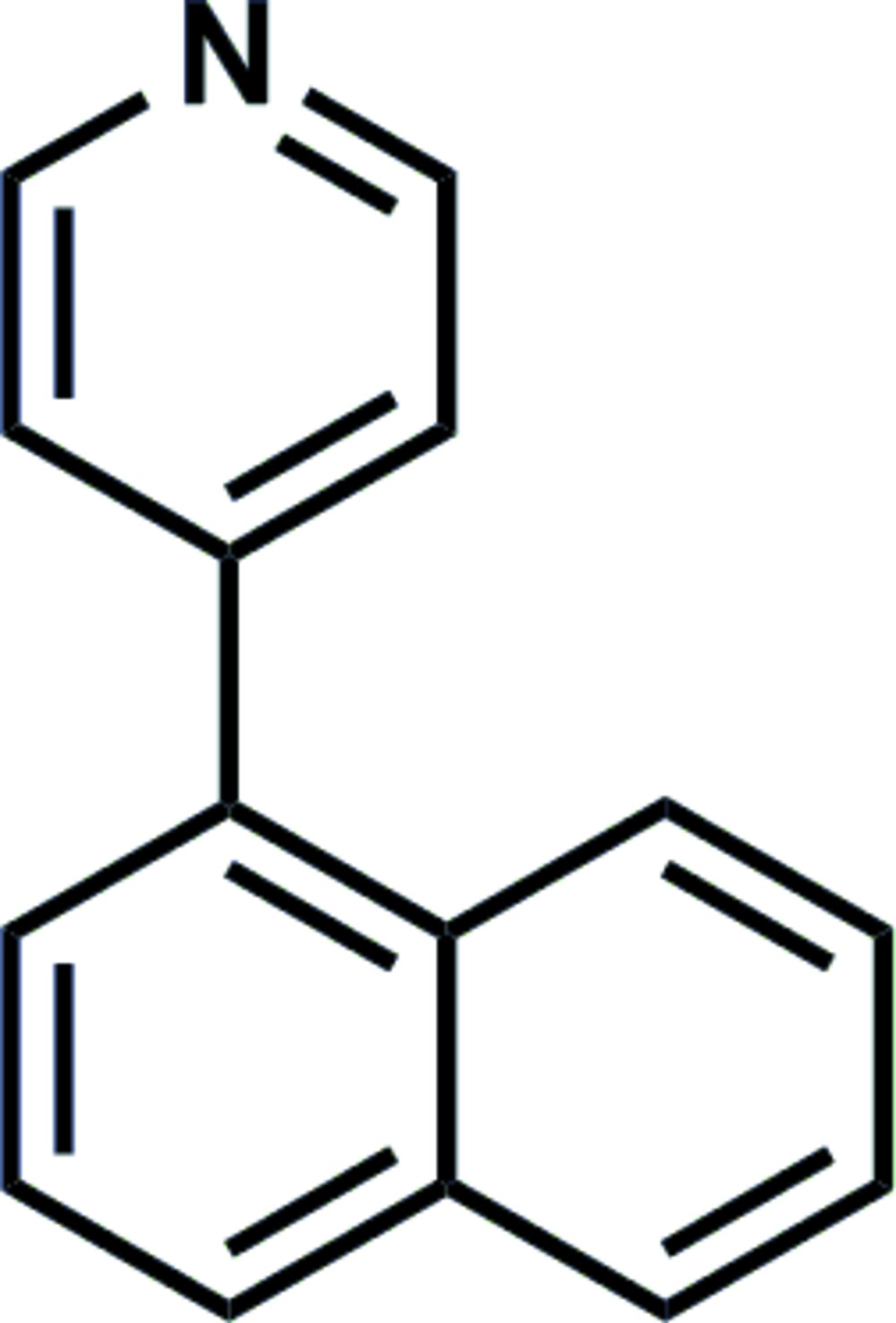


## Experimental

### 

#### Crystal data

C_15_H_11_N
*M* *_r_* = 205.25
Monoclinic, 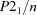

*a* = 6.8487 (2) Å
*b* = 7.4436 (2) Å
*c* = 21.8378 (5) Å
β = 91.833 (1)°
*V* = 1112.70 (5) Å^3^
*Z* = 4
Mo *K*α radiation
μ = 0.07 mm^−1^
*T* = 193 K
0.53 × 0.43 × 0.43 mm

#### Data collection

Bruker X8 APEX CCD diffractometer
14800 measured reflections
2831 independent reflections
2302 reflections with *I* > 2σ(*I*)
*R*_int_ = 0.019

#### Refinement

*R*[*F* ^2^ > 2σ(*F* ^2^)] = 0.047
*wR*(*F* ^2^) = 0.147
*S* = 1.05
2831 reflections
145 parameters
H-atom parameters constrained
Δρ_max_ = 0.25 e Å^−3^
Δρ_min_ = −0.18 e Å^−3^

### 

Data collection: *SMART* (Bruker, 2007 ▶); cell refinement: *SAINT-NT* (Bruker, 2007 ▶); data reduction: *SAINT-NT*; program(s) used to solve structure: *SHELXS97* (Sheldrick, 2008 ▶); program(s) used to refine structure: *SHELXL97* (Sheldrick, 2008 ▶); molecular graphics: *ORTEP-3 for Windows* (Farrugia, 2012 ▶); software used to prepare material for publication: *SHELXTL* (Sheldrick, 2008 ▶).

## Supplementary Material

Crystal structure: contains datablock(s) I, global. DOI: 10.1107/S1600536813014372/fj2629sup1.cif

Structure factors: contains datablock(s) I. DOI: 10.1107/S1600536813014372/fj2629Isup2.hkl

Supplementary material file. DOI: 10.1107/S1600536813014372/fj2629Isup3.cml

Additional supplementary materials:  crystallographic information; 3D view; checkCIF report

## Figures and Tables

**Table 1:** Hydrogen-bond geometry (Å, °) *Cg*1 is the centroid of the C5–C10 ring.

*D*—H⋯*A*	*D*—H	H⋯*A*	*D*⋯*A*	*D*—H⋯*A*
C6—H6⋯*Cg*1^i^	0.93	2.69	3.577 (2)	161
C14—H14⋯*Cg*1^ii^	0.93	2.84	3.648 (2)	146

Symmetry codes: (i) 
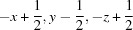
; (ii) 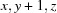.

**Fig. 1.**
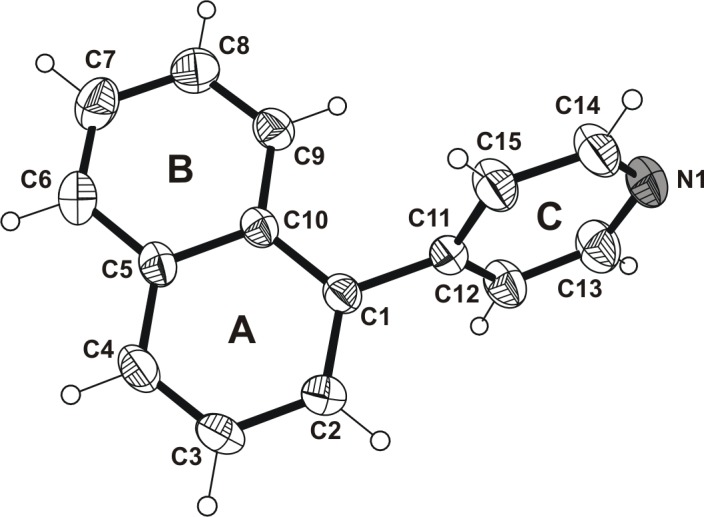
Asymmetric unit of the title compound, showing the atom numbering scheme. Displacement ellipsoids for non-hydrogen atoms are drawn at the 50% probability level.

**Fig. 2.**
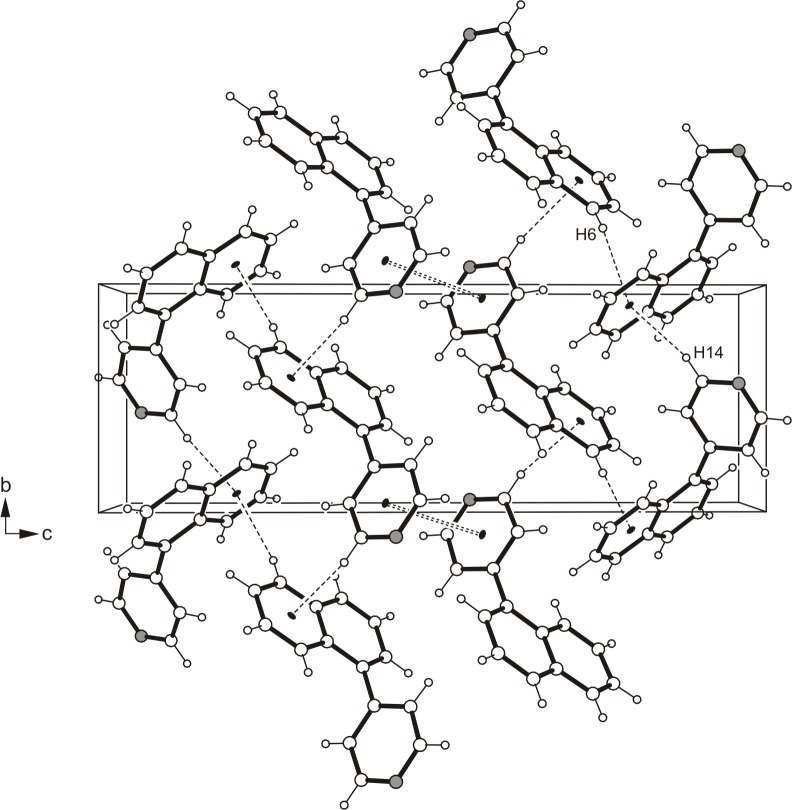
A view along the a-axis of the title compound. Hydrogen bond type contacts are presented as broken, π···π stacking interactions as broken double lines.

## supplementary crystallographic information

### Comment

Molecules having a defined structure with rather predictable supramolecular interactions of their construction elements and functional groups such as π···π (James, 2004) or weak hydrogen bonding contacts (Desiraju & Steiner,1999) are helpful in gaining deeper insight into the principles of crystal engineering (Tiekink *et al.*, 2010). This has stimulated to determine the crystal structure of the title compound being composed of two π-systems of different electronic nature (naphthalene and pyridine units) and having potential capability of weak C—H···π (Nishio *et al.*, 2009) or C—H···N bonding (Desiraju & Steiner, 1999). In the crystal structure, the bond distances both of the naphthalene (AB) and pyridine (C) parts agree well with those found for related compounds (Boeyens *et al.*, (1988) Suthar *et al.*, 2005). The naphthalene moiety shows a slight distortion from strict planarity with largest atomic distances from the best plane being 0.029 (1) Å for C7 and -0.030 (2) Å for C9. The mean planes of the naphthalene and pyridine moieties are inclined to one another by 72.9 (1) ° (Fig. 1). Contrary to expectations, the nitrogen of the heterocyclic ring is excluded from molecular association. Instead, the crystal structure is stabilized by weaker C—H···π contacts with the non-substituted ring of the naphthalene unit (B) acting as an acceptor [C6—H6···centroid(B) 2.69 Å, 161 °, C14—H14···centroid(B) 2.84 Å, 146 °]. Moreover, the centre···centre distance of 3.772 (2) Å between the pyridine rings of inversion related molecules indicate the occurrence of π···π stacking interactions (Fig. 2). In a similar fashion as in the crystal structure of naphthalene (Fabbiani *et al.*, 2006), each molecule is surrounded by another six molecules so that their naphthalene elements form a herringbone motif.

### Experimental

Preparation of the title compound was achieved by a Suzuki cross coupling reaction (Miyaura *et al.*, 1981) between 2-(1-naphthyl)-1,3,2-dioxaborolane (Broutin & Colobert, 2005) (4.94 g, 25 mmol) and 4-bromopyridinium hydrochloride (4.87 g, 25 mmol) in the presence of tetrakis(triphenylphosphane)palladium (0.52 g, 0.45 mmol) and potassium phosphate (7.24 g, 34 mmol) in 136 ml degassed *N,N*-dimethylformamide. The resulting mixture was heated to 100 °C and stirred at this temperature for 6 h. After cooling to room temperature, the mixture was extracted with toluene. The extract was washed with saturated aqueous NaCl solution and dried (Na_2_SO_4_). Evaporation of the solvent and crystallization from ethanol yielded 1.10 g (24%) colourless crystals. M.p. (366–368 K). Spectroscopic data correspond to those reported for the compound obtained *via* a different synthetic route (Molander & Beaumard, 2010).

### Refinement

Aromatic H atoms were positioned geometrically and allowed to ride on their respective parent atoms, with C—H = 0.95 Å and *U*_iso_(H) = 1.2 *U*_eq_(C).

### Crystal data

**Table:**

C_15_H_11_N	*F*(000) = 432
*M**_r_* = 205.25	*D*_x_ = 1.225 Mg m^−^^3^
Monoclinic, *P*2_1_/*n*	Mo *K*α radiation, λ = 0.71073 Å
Hall symbol: -P 2yn	Cell parameters from 7291 reflections
*a* = 6.8487 (2) Å	θ = 2.9–32.2°
*b* = 7.4436 (2) Å	µ = 0.07 mm^−^^1^
*c* = 21.8378 (5) Å	*T* = 193 K
β = 91.833 (1)°	Irregular, colourless
*V* = 1112.70 (5) Å^3^	0.53 × 0.43 × 0.43 mm
*Z* = 4	

### Data collection

**Table:**

Bruker X8 APEX CCD diffractometer	2302 reflections with *I* > 2σ(*I*)
Radiation source: fine-focus sealed tube	*R*_int_ = 0.019
Graphite monochromator	θ_max_ = 28.6°, θ_min_ = 1.9°
φ and ω scans	*h* = −7→9
14800 measured reflections	*k* = −10→9
2831 independent reflections	*l* = −29→28

### Refinement

**Table:**

Refinement on *F*^2^	Primary atom site location: structure-invariant direct methods
Least-squares matrix: full	Secondary atom site location: difference Fourier map
*R*[*F*^2^ > 2σ(*F*^2^)] = 0.047	Hydrogen site location: inferred from neighbouring sites
*wR*(*F*^2^) = 0.147	H-atom parameters constrained
*S* = 1.05	*w* = 1/[σ^2^(*F*_o_^2^) + (0.0714*P*)^2^ + 0.2598*P*] where *P* = (*F*_o_^2^ + 2*F*_c_^2^)/3
2831 reflections	(Δ/σ)_max_ < 0.001
145 parameters	Δρ_max_ = 0.25 e Å^−^^3^
0 restraints	Δρ_min_ = −0.18 e Å^−^^3^

### Special details

**Table:**

Geometry. All e.s.d.'s (except the e.s.d. in the dihedral angle between two l.s. planes) are estimated using the full covariance matrix. The cell e.s.d.'s are taken into account individually in the estimation of e.s.d.'s in distances, angles and torsion angles; correlations between e.s.d.'s in cell parameters are only used when they are defined by crystal symmetry. An approximate (isotropic) treatment of cell e.s.d.'s is used for estimating e.s.d.'s involving l.s. planes.
Refinement. Refinement of *F*^2^ against ALL reflections. The weighted *R*-factor *wR* and goodness of fit *S* are based on *F*^2^, conventional *R*-factors *R* are based on *F*, with *F* set to zero for negative *F*^2^. The threshold expression of *F*^2^ > σ(*F*^2^) is used only for calculating *R*-factors(gt) *etc*. and is not relevant to the choice of reflections for refinement. *R*-factors based on *F*^2^ are statistically about twice as large as those based on *F*, and *R*-factors based on ALL data will be even larger.

### Fractional atomic coordinates and isotropic or equivalent isotropic displacement parameters (Å^2^)

**Table:**

	*x*	*y*	*z*	*U*_iso_*/*U*_eq_	
C1	0.12887 (17)	0.13749 (16)	0.10279 (5)	0.0377 (3)	
C2	0.2963 (2)	0.1121 (2)	0.07098 (6)	0.0502 (3)	
H2	0.3218	0.1859	0.0378	0.060*	
C3	0.4299 (2)	−0.0245 (2)	0.08800 (7)	0.0568 (4)	
H3	0.5416	−0.0412	0.0656	0.068*	
C4	0.3968 (2)	−0.13166 (19)	0.13686 (7)	0.0517 (3)	
H4	0.4867	−0.2206	0.1477	0.062*	
C5	0.22727 (18)	−0.10975 (16)	0.17143 (6)	0.0405 (3)	
C6	0.1913 (2)	−0.21677 (19)	0.22348 (7)	0.0521 (3)	
H6	0.2814	−0.3045	0.2354	0.063*	
C7	0.0282 (2)	−0.1939 (2)	0.25625 (7)	0.0587 (4)	
H7	0.0089	−0.2637	0.2908	0.070*	
C8	−0.1115 (2)	−0.0647 (2)	0.23796 (7)	0.0552 (4)	
H8	−0.2247	−0.0516	0.2600	0.066*	
C9	−0.08234 (18)	0.04175 (17)	0.18808 (6)	0.0433 (3)	
H9	−0.1765	0.1262	0.1764	0.052*	
C10	0.08913 (16)	0.02542 (15)	0.15386 (5)	0.0356 (3)	
C11	−0.00895 (17)	0.28518 (16)	0.08512 (5)	0.0383 (3)	
C12	−0.1289 (2)	0.27550 (19)	0.03312 (6)	0.0519 (3)	
H12	−0.1247	0.1757	0.0076	0.062*	
C13	−0.2554 (2)	0.4160 (2)	0.01943 (7)	0.0603 (4)	
H13	−0.3365	0.4058	−0.0154	0.072*	
C14	−0.1506 (2)	0.5733 (2)	0.10206 (8)	0.0595 (4)	
H14	−0.1552	0.6763	0.1261	0.071*	
C15	−0.0213 (2)	0.43915 (19)	0.11996 (7)	0.0537 (4)	
H15	0.0569	0.4526	0.1553	0.064*	
N1	−0.26865 (18)	0.56431 (17)	0.05271 (6)	0.0572 (3)	

### Atomic displacement parameters (Å^2^)

**Table:**

	*U*^11^	*U*^22^	*U*^33^	*U*^12^	*U*^13^	*U*^23^
C1	0.0399 (6)	0.0367 (6)	0.0362 (5)	0.0064 (4)	−0.0017 (4)	−0.0047 (4)
C2	0.0521 (8)	0.0563 (8)	0.0428 (6)	0.0127 (6)	0.0091 (6)	0.0022 (6)
C3	0.0486 (8)	0.0686 (9)	0.0540 (8)	0.0212 (7)	0.0123 (6)	−0.0027 (7)
C4	0.0460 (7)	0.0516 (8)	0.0571 (8)	0.0194 (6)	−0.0029 (6)	−0.0033 (6)
C5	0.0402 (6)	0.0373 (6)	0.0435 (6)	0.0048 (5)	−0.0073 (5)	−0.0031 (5)
C6	0.0531 (8)	0.0455 (7)	0.0569 (8)	0.0037 (6)	−0.0104 (6)	0.0099 (6)
C7	0.0618 (9)	0.0573 (9)	0.0568 (8)	−0.0067 (7)	−0.0005 (7)	0.0170 (7)
C8	0.0471 (8)	0.0601 (8)	0.0589 (8)	−0.0050 (6)	0.0105 (6)	0.0056 (7)
C9	0.0369 (6)	0.0428 (6)	0.0504 (7)	0.0027 (5)	0.0015 (5)	−0.0007 (5)
C10	0.0350 (6)	0.0331 (5)	0.0383 (6)	0.0021 (4)	−0.0043 (4)	−0.0050 (4)
C11	0.0381 (6)	0.0386 (6)	0.0383 (6)	0.0045 (5)	0.0027 (4)	0.0011 (4)
C12	0.0523 (8)	0.0491 (7)	0.0536 (7)	0.0098 (6)	−0.0100 (6)	−0.0085 (6)
C13	0.0536 (8)	0.0660 (9)	0.0603 (8)	0.0133 (7)	−0.0156 (7)	0.0007 (7)
C14	0.0652 (9)	0.0452 (7)	0.0681 (9)	0.0169 (7)	−0.0011 (7)	−0.0076 (7)
C15	0.0608 (9)	0.0479 (7)	0.0516 (7)	0.0134 (6)	−0.0114 (6)	−0.0087 (6)
N1	0.0518 (7)	0.0529 (7)	0.0669 (8)	0.0175 (5)	−0.0010 (6)	0.0064 (6)

### Geometric parameters (Å, º)

**Table:**

C1—C2	1.3723 (17)	C8—C9	1.367 (2)
C1—C10	1.4259 (17)	C8—H8	0.9300
C1—C11	1.4916 (16)	C9—C10	1.4172 (17)
C2—C3	1.4098 (19)	C9—H9	0.9300
C2—H2	0.9300	C11—C15	1.3797 (18)
C3—C4	1.357 (2)	C11—C12	1.3823 (17)
C3—H3	0.9300	C12—C13	1.3849 (19)
C4—C5	1.4144 (19)	C12—H12	0.9300
C4—H4	0.9300	C13—N1	1.326 (2)
C5—C6	1.4161 (19)	C13—H13	0.9300
C5—C10	1.4252 (16)	C14—N1	1.328 (2)
C6—C7	1.356 (2)	C14—C15	1.3828 (19)
C6—H6	0.9300	C14—H14	0.9300
C7—C8	1.405 (2)	C15—H15	0.9300
C7—H7	0.9300		
			
C2—C1—C10	119.92 (11)	C7—C8—H8	119.7
C2—C1—C11	120.16 (11)	C8—C9—C10	121.00 (12)
C10—C1—C11	119.90 (10)	C8—C9—H9	119.5
C1—C2—C3	120.78 (13)	C10—C9—H9	119.5
C1—C2—H2	119.6	C9—C10—C5	118.21 (11)
C3—C2—H2	119.6	C9—C10—C1	122.98 (10)
C4—C3—C2	120.51 (13)	C5—C10—C1	118.81 (11)
C4—C3—H3	119.7	C15—C11—C12	116.81 (12)
C2—C3—H3	119.7	C15—C11—C1	121.27 (11)
C3—C4—C5	120.82 (12)	C12—C11—C1	121.92 (11)
C3—C4—H4	119.6	C11—C12—C13	119.29 (13)
C5—C4—H4	119.6	C11—C12—H12	120.4
C4—C5—C6	122.03 (12)	C13—C12—H12	120.4
C4—C5—C10	119.14 (11)	N1—C13—C12	124.30 (14)
C6—C5—C10	118.83 (12)	N1—C13—H13	117.8
C7—C6—C5	121.34 (13)	C12—C13—H13	117.8
C7—C6—H6	119.3	N1—C14—C15	124.12 (14)
C5—C6—H6	119.3	N1—C14—H14	117.9
C6—C7—C8	120.05 (13)	C15—C14—H14	117.9
C6—C7—H7	120.0	C11—C15—C14	119.60 (13)
C8—C7—H7	120.0	C11—C15—H15	120.2
C9—C8—C7	120.51 (13)	C14—C15—H15	120.2
C9—C8—H8	119.7	C13—N1—C14	115.86 (12)
			
C10—C1—C2—C3	0.3 (2)	C2—C1—C10—C9	−178.79 (12)
C11—C1—C2—C3	178.62 (13)	C11—C1—C10—C9	2.86 (17)
C1—C2—C3—C4	−1.1 (2)	C2—C1—C10—C5	1.02 (17)
C2—C3—C4—C5	0.5 (2)	C11—C1—C10—C5	−177.32 (10)
C3—C4—C5—C6	−178.45 (14)	C2—C1—C11—C15	−105.67 (15)
C3—C4—C5—C10	0.8 (2)	C10—C1—C11—C15	72.68 (16)
C4—C5—C6—C7	179.85 (14)	C2—C1—C11—C12	73.83 (17)
C10—C5—C6—C7	0.6 (2)	C10—C1—C11—C12	−107.83 (14)
C5—C6—C7—C8	1.5 (2)	C15—C11—C12—C13	−1.2 (2)
C6—C7—C8—C9	−1.6 (2)	C1—C11—C12—C13	179.30 (13)
C7—C8—C9—C10	−0.3 (2)	C11—C12—C13—N1	1.1 (3)
C8—C9—C10—C5	2.34 (18)	C12—C11—C15—C14	0.4 (2)
C8—C9—C10—C1	−177.84 (12)	C1—C11—C15—C14	179.90 (13)
C4—C5—C10—C9	178.26 (11)	N1—C14—C15—C11	0.6 (3)
C6—C5—C10—C9	−2.44 (17)	C12—C13—N1—C14	−0.1 (2)
C4—C5—C10—C1	−1.57 (17)	C15—C14—N1—C13	−0.8 (2)
C6—C5—C10—C1	177.74 (11)		

### Hydrogen-bond geometry (Å, º)

Cg1 is the centroid of the C5–C9 ring.

**Table:**

*D*—H···*A*	*D*—H	H···*A*	*D*···*A*	*D*—H···*A*
C6—H6···*Cg*1^i^	0.93	2.69	3.577 (2)	161
C14—H14···*Cg*1^ii^	0.93	2.84	3.648 (2)	146

Symmetry codes: (i) −*x*+1/2, *y*−1/2, −*z*+1/2; (ii) *x*, *y*+1, *z*.
